# A novel method to realize a non-uniform heat flux distribution through the variable-speed scanning of an electron beam

**DOI:** 10.1038/s41598-021-92730-x

**Published:** 2021-06-24

**Authors:** Chuanmao Zheng, Hongxin Yao, Xiyao Wang, Hong Ye

**Affiliations:** 1 Department of Thermal Science and Energy Engineering, University of Science and Technology of China, Hefei, 230026 China; 2 China Aerodynamics Research and Development Center, Mianyang, 621000 China

**Keywords:** Energy science and technology, Engineering

## Abstract

Quartz lamp heaters and hypersonic wind tunnel are currently applied in thermal assessment of heat resistant materials and surface of aircraft. However, it is difficult to achieve precise heat flux distribution by quartz lamp heaters, while enormous energy is required by a large scale hypersonic wind tunnel. Electron beam can be focused into a beam spot of millimeter scale by an electromagnetic lens and electron-magnetically deflected to achieve a rapid scanning over a workpiece. Moreover, it is of high energy utilization efficiency when applying an electron beam to heat a metal workpiece. Therefore, we propose to apply an electron beam with a variable speed to establish a novel method to realize various non-uniform heat flux boundary conditions. Besides, an electron beam thermal assessment equipment is devised. To analyze the feasibility of this method, an approach to calculate the heat flux distribution formed by an electron beam with variable-speed scanning is constructed with beam power, diameter of the beam spot and dwell duration of the electron beam at various locations as the key parameters. To realize a desired non-uniform heat flux distribution of the maximum gradient of 1.1 MW/m^3^, a variable-speed scanning strategy is constructed on basis of the conservation of energy. Compared with the desired heat flux, the maximum deviation of the scanned heat flux is 4.5% and the deviation in the main thermal assessment area is less than 3%. To verify the method, taking the time-average scanned heat flux as the boundary condition, a heat transfer model is constructed and temperature results are calculated. The experiment of variable-speed scanning of an electron beam according to the scanning strategy has been carried out. The measured temperatures are in good agreement with the predicted results at various locations. Temperature fluctuation during the scanning process is analyzed, and it is found to be proportional to the scanned heat flux divided by volumetric heat capacity, which is applicable for different materials up to 3.35 MW/m^2^. This study provides a novel and effective method for precise realization of various non-uniform heat flux boundary conditions.

## Introduction

High heat flux ground tests have been widely applied in thermal assessment of heat resistant materials^1^ and hypersonic aircraft^2,3^, and the most common test apparatuses include hypersonic arc wind tunnel and quartz lamp heaters^4–6^. Hypersonic arc wind tunnel can provide high enthalpy gas for hundreds of seconds^7^, thus it is able to maintain long-duration experimental conditions for heat resistent materials. Anuscheh Nawaz et al.^8^ conducted arc jet tests with different enthalpy values from 5.7 to 22.9 MJ/kg, and the measured heat flux at the stagnation point ranged from 0.57 to 4.35 MW/m^2^. To test ablation resistance performance of ZrB2-SiC ceramic materials, S. Mungiguerra et al.^9^ conducted arc tunnel tests under the conditions of Mach number of 2.6 and enthalpy value of 20 MJ/kg, in which the highest temperature in the leading edge exceeded 2400 K. However, a large scale arc wind tunnel requires enormous energy and the construction cost is extremely high. By contrast, the cost of the quartz lamp heaters is relatively lower, and it has been developed into a well-established technology^4,10^. Quartz lamp heaters are composed of multiple quartz lamps, reflectors and baffles. Quartz lamps emit thermal radiation, and reflectors and baffles can improve energy efficiency and control the heating range. In the 1980s, analytical methods were used to calculate the heat flux distribution of a single quartz lamp^11,12^, but these methods were incapable of predicting the heat flux distribution formed by multiple quartz lamps. Besides, the influences of the reflectors and baffles were not considered. To this end, many scholars have performed related works in the aspects of simulation and experiment. Turner et al. constructed a photon trajectory tracking model of radiation from quartz lamp heaters using the Monte Carlo method, and verified the simulated results by experiments^13^. Ziemke et al.^14^ analyzed the heat flux distribution of quartz lamp heaters by a finite element method, and achieved uniform heat flux by adjusting the position and the power of each quartz lamp. NASA Dryden Flight Research Center has built a quartz lamp heating platform with the maximum power of 3.5 MW, which can achieve the maximum heat flux of 1.1 MW/m^2^ and the highest temperature of 1900 K^10^. On the platform, Jenkins et al. performed a thermal assessment experiment of non-uniform temperature distribution on the YF-12A high-speed aircraft by dividing it into several zones, and the zones were heated individually by quartz heaters of different power^15^. However, the boundary lines of the zones may be overheated by multiple quartz lamps or insufficiently heated, and the deviation of the heat flux from the desired value can reach 10% or even higher^16^. Therefore, it is difficult for achieve a precise non-uniform heat flux distribution by the quartz lamp heaters. Moreover, the heat flux of the leading edge of hypersonic aircrafts^5^ can reach 1.5 MW/m^2^, which is beyond the capacity of quartz lamp heaters.

An electron beam can be focused into a beam spot of millimeter scale with an energy density of 10^3^ MW/m^2^^17^ by an electromagnetic lens and electronmagetically deflected to achieve a rapid scanning over a workpiece^18^. Thus it has been widely applied in welding^19,20^ of metal materials and 3D printing of complex workpieces^21,22^. Moreover, an electron beam can directly interact with materials, and the energy efficiency of welding metal materials is generally among 0.7–0.9^23,24^. Wentao Yan^25^ has done a simulation of a large number of electrons of the acceleration voltage of 60 kV interacting with Ti–6Al–4V by an open-source code CASINO^26^. The principle of CASINO is to simulate the trajectories and energy loss of electrons during the interaction with atoms of the workpiece by the Monte Carlo method. The absorbed energy and back-scattered energy can be obtained through the simulation, and the absorption efficiency is 0.90. Therefore, an electron beam has the advantages of high energy density, high resolution and high energy efficiency. Hence we propose a novel method to apply an electron beam as the heat source to realize non-uniform precise heat flux boundary conditions over a workpiece by variable-speed scanning of high frequency, which can be applied in the thermal assessment of heat resistant materials and hypersonic aircraft.

In this study, a calculation approach of heat flux distribution formed by a scanning electron beam is established. To realize a desired non-uniform heat flux distribution, a scanning strategy is constructed based on the conservation of energy. The calculated scanned heat flux is compared with the desired heat flux. Temperature fluctuation during the scanning process is analyzed at various locations. A heat transfer model with a boundary condition of the desired heat flux is constructed and temperature results are calculated. The experiment of variable-speed scanning of an electron beam according to the scanning strategy has been carried out. Temperatures of simulation and experiment at various locations are compared to examine the performance of the variable-speed scanning method.

## Model and experiment

### The method of variable-speed scanning

In the scanning process, the electron moves with variable speed. The advantage is to produce a pseudo-large beam with an arbitrary shape as a time average, i.e., a non-uniform heat flux. Therefore, various heat flux distributions can be obtained by changing the dwell durations in the program. The method can be summarized in 4 steps. Firstly, the desired heat flux and the workpiece are prepared. Secondly, divide the scanning area of the workpiece into a large number of elements and determine the locations of them. Thirdly, construct the calculation formulas of the time-average heat flux of the elements with beam power, diameter of the beam spot and dwell duration. Finally, obtain the scanning strategy according to the desired heat flux.

**Figure 1:**
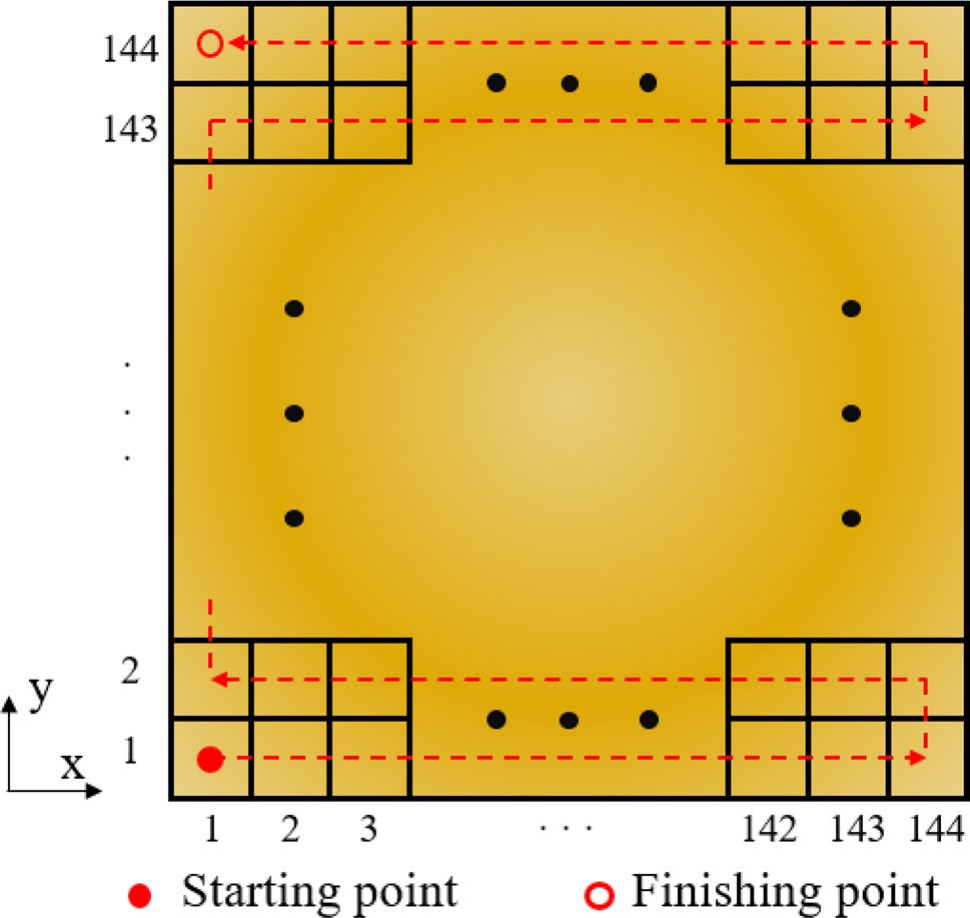
Schematic of the scan path.

The dimension of the workpiece is 360 mm × 360 mm × 6 mm, and the top surface of the workpiece is the scanning area. To hit the whole scanning area with an electron beam, a large number of elements are required. The scanning area is evenly divided into 144 parts in both the x and y directions, thus there are 20,736 elements in total. The scan step is 2.5 mm and the area of each element is denoted by *S*. The scan path passes through the center of each element, as shown in Fig. 1. In a scanning cycle, the electron beam sets out from the starting point, jumps successively along the scan path until it reaches the finishing point. The serial number of each element is marked from 1 to 20,736 along the scan path, and the central coordinate of the i-th element is denoted by $\left( {x_{{\text{i}}} ,y_{{\text{i}}} } \right)$. It is assumed that the center of a beam spot coincides with the center of each element, therefore, the position of the beam spot can be described by the coordinates of the elements. Dwell duration of the center of a beam spot in the element i is denoted by $\Delta t_{{\text{i}}}$, and the total dwell duration in all the elements can be calculated as $\sum\nolimits_{{{\text{i}} = 1}}^{N} {\Delta t_{{\text{i}}} } = T_{0} \left( {N = 20736} \right)$, where *T*_0_ is the duration in a cycle. In the following analysis and experiment, *T*_0_ is set as a constant.

When the center of a beam spot is located in the element i, the transient heat flux boundary formed on an arbitrary element j can be denoted by $q^{\prime\prime}_{{{\text{ij}}}} \left( {x_{{\text{j}}} ,y_{{\text{j}}} } \right)$. As the heat flux of an electron beam can be described by the Gaussian surface heat source model^27^, $q^{\prime\prime}_{{{\text{ij}}}} \left( {x_{{\text{j}}} ,y_{{\text{j}}} } \right)$ can be expressed as

1 $$q^{\prime\prime}_{{{\text{ij}}}} \left( {x_{{\text{j}}} ,y_{{\text{j}}} } \right) = \frac{{3P}}{{\pi R^{2} }}{\text{exp}}\left[ { - 3\frac{{\left( {x_{{\text{j}}} - x_{{\text{i}}} } \right)^{2} + \left( {y_{{\text{j}}} - y_{{\text{i}}} } \right)^{2} }}{{R^{2} }}} \right]$$

where *P* is the beam power, and *R* is the radius of the beam spot. The heat flux within the beam spot is far higher than that outside.

To calculate the heat flux distribution on the top surface of the workpiece, total heat allocated to the element j in a cycle is $\sum\nolimits_{{{\text{i}} = 1}}^{N} {q^{\prime\prime}_{{{\text{ij}}}} \left( {x_{{\text{j}}} ,y_{{\text{j}}} } \right)S\Delta t_{{\text{i}}} }$. Hence the time-average scanned heat flux of the element j can be defined as

2 $$\overline{{q^{\prime\prime}}} \left( {x_{{\text{j}}} ,y_{{\text{j}}} } \right) = \sum\limits_{{{\text{i}} = 1}}^{N} {q^{\prime\prime}_{{{\text{ij}}}} \left( {x_{{\text{j}}} ,y_{{\text{j}}} } \right)\Delta t_{{\text{i}}} } /T_{0}$$

If a desired non-uniform heat flux is denoted as $Q^{\prime\prime}_{0} \left( {x,y} \right)$, the desired heat flux in an arbitrary element j can be expressed as $Q^{\prime\prime}_{0} \left( {x_{{\text{j}}} ,y_{{\text{j}}} } \right)$, and the desired beam power is *P*_0_. According to the principle of energy conservation, the desired heat flux should be consistent with the time-average scanned heat flux given in Eq. (2), i.e.:

3 $$Q^{\prime\prime}_{0} \left( {x_{{\text{j}}} ,y_{{\text{j}}} } \right) = \overline{{q^{\prime\prime}}} \left( {x_{{\text{j}}} ,y_{{\text{j}}} } \right)$$

Incorporating Eq. (1) and Eq. (2) into Eq. (3), we have

4 $$Q^{\prime\prime}_{0} \left( {x_{{\text{j}}} ,y_{{\text{j}}} } \right)T_{0} = \frac{{3P_{0} }}{{\pi R^{2} }}\sum\limits_{{{\text{i}} = 1}}^{N} {{\text{exp}}\left[ { - 3\frac{{\left( {x_{{\text{j}}} - x_{{\text{i}}} } \right)^{2} + \left( {y_{{\text{j}}} - y_{{\text{i}}} } \right)^{2} }}{{R^{2} }}} \right]\Delta t_{{\text{i}}} }$$

Through Eq. (4), it can be seen that to realize the desired heat flux, the key parameters include the beam power, the diameter of the beam spot and the dwell duration. The beam power and the diameter of the beam spot can be set as constants. By solving the equation set composed of Eq. (4) for all the elements, the dwell durations can be obtained. Consequently, the scanning strategy can be determined.

### Experimental system

**Figure 2:**
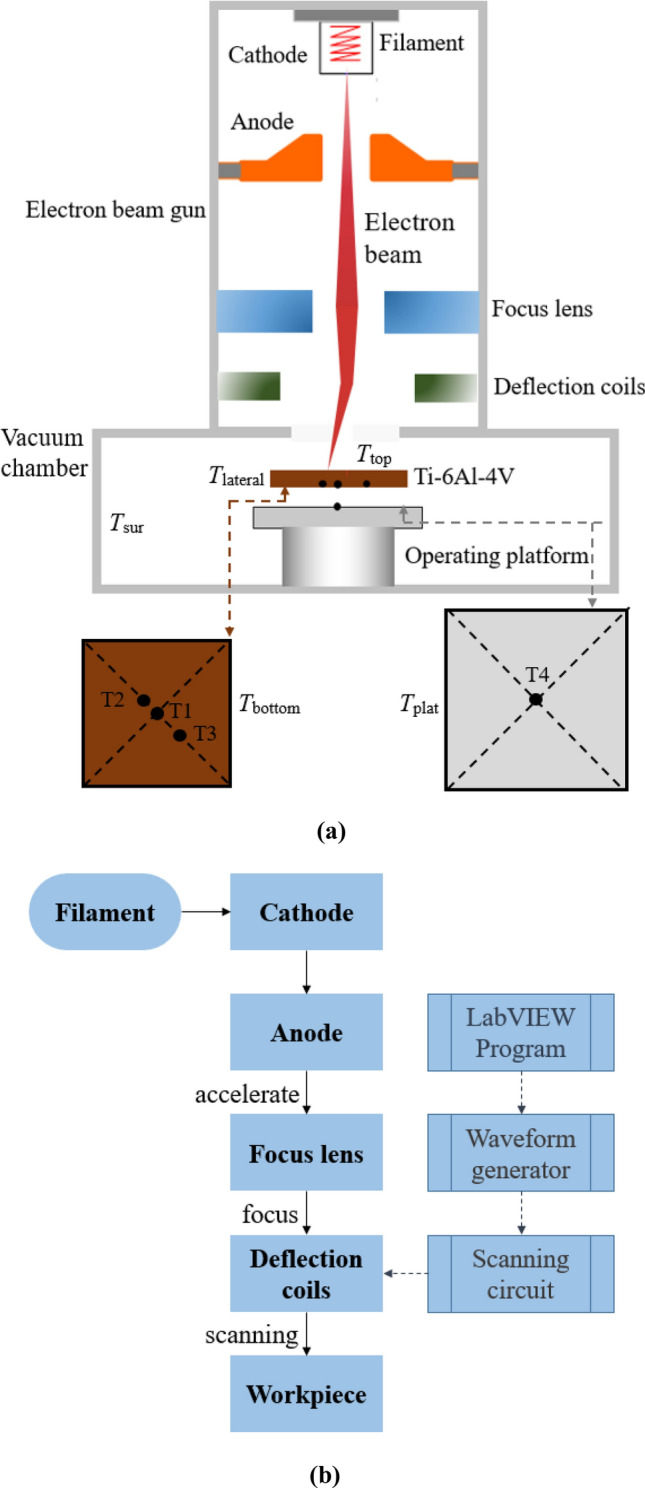
Schematic of the experimental equipment and the beam controlling scheme. (**a**) Schematic of the electron beam thermal assessment equipment. (**b**) Schematic of the controlling scheme and formation process of an electron beam.

**Figure 3:**
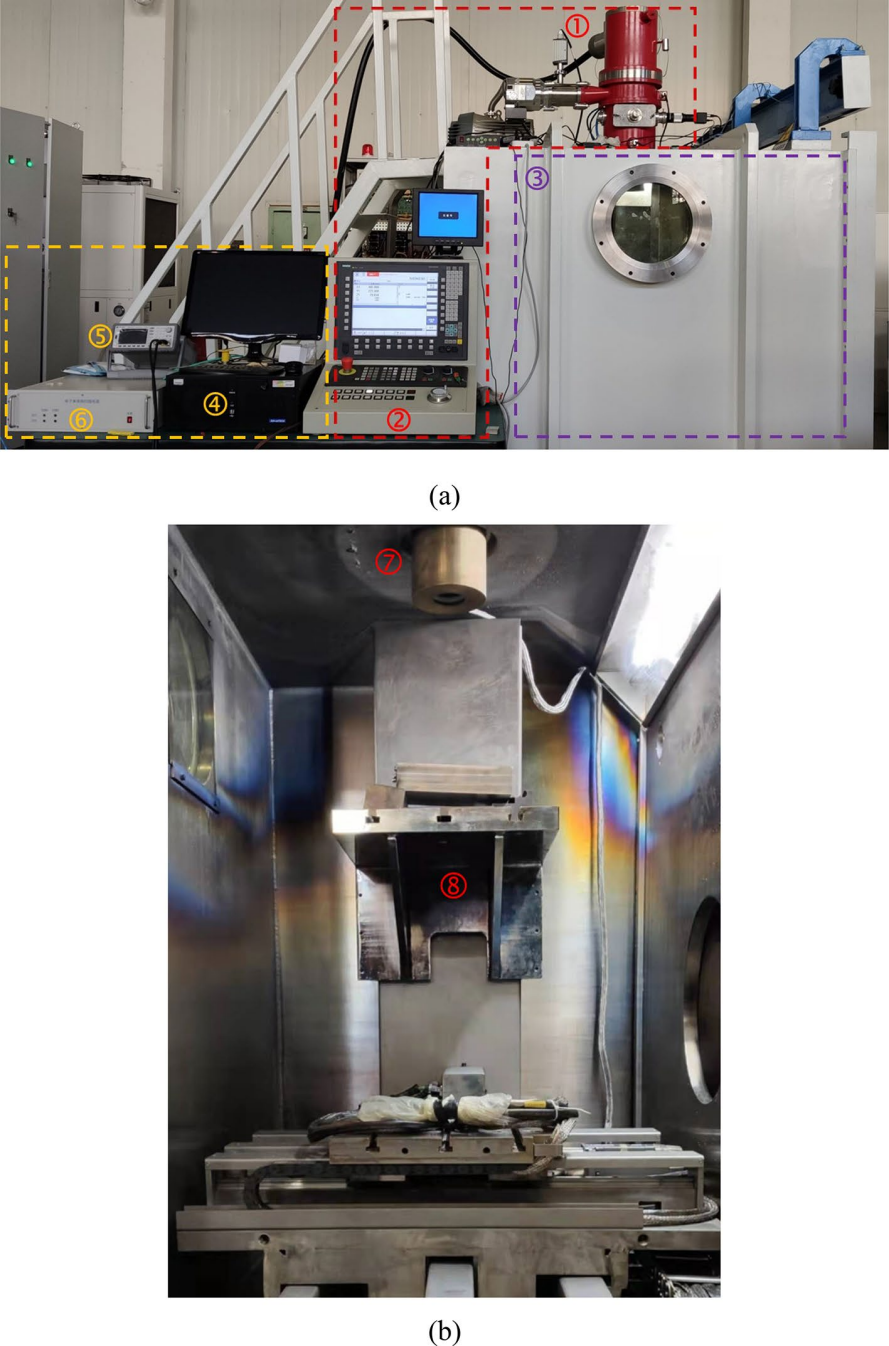
The photos of the equipment setup. (**a**) The components of the equipment setup. (**b**) The components inside the vacuum chamber. ➀ Electron beam gun. ➁ The beam control system. ➂ Vacuum chamber. ➃ PC with the LabVIEW program. ➄ Waveform generator. ➅ Scan drive circuit. ➆ Helmholtz coils. ➇ Platform.

Figure 2a shows the schematic of the electron beam thermal assessment equipment, which consists of a tungsten filament, a cathode, an anode, focus lens, Helmholtz deflection coils with the control system, a vacuum chamber and an operation platform. The accelerating voltage between the cathode and anode can be up to 60 kV. The Helmholtz deflection coils can provide a magnetic field with the maximum frequency of 100 kHz, namely the electron beam can jump 100 thousand times in 1 s. The controlling scheme is composed of a LabVIEW program, a KEYSIGHT 33500b waveform generator and a high frequency scan drive circuit, as Fig. 2b shows. Firstly, the scan path and dwell duration are both set in the LabVIEW program, and the dwell duration can be set in the range of 1 μs to *T*_0_ according to needs, where 1 μs is the shortest duration owing to the inductance of the Helmholtz coils and *T*_0_ is the duration in a cycle. Then the signal is transferred to the waveform generator and the scan drive circuit in order to generate the voltage signal of high frequency. Finally, the voltage signal is transferred to the Helmholtz deflecting coils and the deflecting magnetic field of high frequency is generated. A proportional-integral-derivative (PID) closed-loop control is applied in the scan drive circuit of the Helmholtz coils. In the system, electrons are generated from the filament and accelerated as an electron beam in the electric field formed by the anode and cathode, and the electron beam is focused electronmagnetically into a beam of high energy density by the focus lens. In the deflecting magnetic field, the beam of high scanning frequency is produced and hit the workpiece. Figure 3a shows the photo of the experiment setup, and Fig. 3b shows the components inside the vacuum chamber. The vacuum chamber is 1.68 m × 1.16 m × 1.85 m in volume, inside which the TC4 workpiece is suspended above the operation platform with no contact. The temperature of the inner surface of the vacuum chamber are denoted by *T*_sur_, the temperatures of the top surface, lateral surface and bottom surface are denoted by *T*_top_, *T*_lateral_ and *T*_bottom_ respectively, the temperature of the top surface of the platform is denoted by *T*_plat_.

In the experiment, the diameter of the beam was set as the maximum size of 5 mm, which is twice the scan step. In this condition, all the area in the top surface of the workpiece can be heated by the electron beam. The scanning frequency was set as 100 kHz, as the number of the elements is 20,736, hence the value of *T*_0_ was 0.2 s.

To measure the temperature distribution of the workpiece, 3 K-type thermocouples marked as T1–T3 were attached to the back of the workpiece to prevent direct interaction with the electron beam, as shown in Fig. 2a T1 was at the center, T2 and T3 were fixed on the diagonal of the plate, the distances from T2 and T3 to the center were 60 and 120 mm individually. The measured *T*_bottom_ at the location of T1–T3 can be obtained. In addition, to measure the surrounding temperature of the bottom surface of the workpiece, 1 K-type thermocouple marked as T4 was attached to the center of the top surface of the operation platform, *T*_plat_ is obtained at the location of T4. Each sensor was scanned at 1-s intervals, and the data were stored in a computer by a data acquisition system. As the temperature of the operation platform kept increasing in the heating process, the surrounding temperatures changed successively. Therefore, when the temperature increasing rate was below 0.05 K/s, it was considered as a stable state.

### Thermal model

To verify the variable-speed scanning method illustrated in “The method of variable-speed scanning” section, experiment and simulation are conducted and the results are compared. The heat sources of the experiment and the simulation are the scanning electron beam and the time-average scanned heat flux formed by the electron beam respectively. Thus the corresponding thermal model requires to be constructed. In addition, the scanning of the electron beam along the scan path results in successive changes of the heat flux distribution at constant intervals on the top surface of the workpiece. Therefore, an instantaneous temperature increment and periodic temperature fluctuation occurs on the top surface, and it decreases along the thickness direction. When the temperature increment and the amplitude of the temperature fluctuation are too large, they may result in great thermal shock and thermal fatigue^28^.

To verify the method and calculate the temperature fluctuation of the TC4 workpiece in the scanning process, a heat transfer model is constructed. The workpiece is in the vacuum surroundings as shown in Fig. 2a, and its top surface is heated by the electron beam. The top surface and lateral surfaces are exposed to the inner surfaces of the equipment whose temperature is denoted by *T*_sur_, which is assumed to be 300 K. The bottom surface is exposed to the top surface of the operation platform. As thermal conduction occurs inside the workpiece, the heat conduction equation can be expressed as

5 $$\nabla \cdot \left( {k\nabla T} \right) = \rho c_{p} \frac{{\partial T}}{{\partial t}}$$

where *ρ*, *k* and *c*_p_ are density, thermal conductivity and specific heat of TC4, respectively. The temperature-dependent thermal properties are listed in Table 1^29^. The top surface gains heat from the surface heat source denoted as $Q^{\prime\prime}\left( {x,y} \right)$.The boundary condition of it can be described as

6 $$q^{\prime\prime}_{{{\text{top}}}} = \eta Q^{\prime\prime}\left( {x,y} \right) - \varepsilon \sigma \left( {T_{{{\text{top}}}}^{4} - T_{{{\text{sur}}}}^{4} } \right)$$

where *η* is the absorption efficiency of Ti-6Al-4 V (TC4) to the electron beam which can be assumed to be 0.90 ^25^ as the acceleration voltage maintained 60 kV in the experiment, *σ* and *ε* are the Stefan-Boltzmann constant and the emissivity given in Table 2^30^ respectively. When the model is applied to verify the method, $Q^{\prime\prime}\left( {x,y} \right)$ refers a time-average scanned heat flux. When the model is applied to calculate the temperature fluctuation, $Q^{\prime\prime}\left( {x,y} \right)$ refers to the heat flux of the scanning electron beam, i.e., $q^{\prime\prime}_{{{\text{ij}}}} \left( {x_{{\text{j}}} ,y_{{\text{j}}} } \right)$ in Eq. (1). The lateral surfaces and the bottom surface lose heat through radiation, hence their boundary conditions can be described as

7 $$q^{\prime\prime}_{{{\text{lateral}}}} = \varepsilon \sigma \left( {T_{{{\text{lateral}}}}^{4} - T_{{{\text{sur}}}}^{4} } \right)$$

and

8 $$q^{\prime\prime}_{{{\text{bottom}}}} = \varepsilon \sigma \left( {T_{{{\text{bottom}}}}^{4} - T_{{{\text{plat}}}}^{4} } \right)$$

respectively, and *T*_plat_ is assumed to be the measured temperature of T4 in the experiment. The model is divided into 415,000 hexahedral grids, and the size of each grid is 2.5 mm × 2.5 mm ×  0.3 mm.

**Table 1:** Thermo-physical properties of TC4.

*T* (K)	*ρ* (kg/m^3^)	*c*_p_ (J/kg K)	*k* (W/m K)
298	4420	546	7.00
373	4406	562	7.45
473	4395	583	8.75
573	4381	606	10.15
673	4366	629	11.35
773	4350	651	12.60
873	4336	673	14.20
973	4324	694	15.50
1073	4309	714	17.80
1173	4294	734	20.20
1268	4271	752	22.70
1268	4271	643	19.2
1373	4267	660	21
1573	4240	696	23.7
1873	4198	750	27

**Table 2:** Emissivity of TC4.

*T*(K)	*ε*
373	0.148
506	0.320
561	0.350
613	0.373
658	0.389
701	0.398
750	0.404
799	0.404
849	0.461
900	0.507
950	0.528
1000	0.531
1023	0.527
1102	0.506
1183	0.466

## Results and discussion

In “The scanning strategy” section, a desired non-uniform heat flux distribution of the maximum gradient of 1.1 MW/m^3^ is designed, and the scanning strategy is obtained by solving the equation set composed of Eq. (4). Time-average scanned heat flux is calculated based on the strategy, which is compared to the desired heat flux to verify the theoretical reliability. In “Verification of the variable-speed scanning method” section, based on the scanning strategy obtained from “The scanning strategy” section, an experiment of three heating stages was conducted to verify the feasibility of the method. In “Temperature fluctuation” section, the amplitude of the temperature fluctuations of the TC4 workpiece and a 304 stainless steel workpiece are investigated to ensure that the method will not cause serious thermal fatigue.

### The scanning strategy

The desired heat flux distribution of the TC4 workpiece is designed as

9 $$Q^{\prime\prime}_{0} \left( {x,y} \right) = \left\{ {\begin{array}{*{20}l} {Q^{\prime\prime}_{{\max }} \cdot \exp \left[ { - 3\frac{{\left( {x - x_{0} } \right)^{2} + \left( {y - y_{0} } \right)^{2} }}{{100^{2} }}} \right]{\text{ ,}} } \hfill & {\sqrt {\left( {x - x_{0} } \right)^{2} + \left( {y - y_{0} } \right)^{2} } {\text{ < 100 }}\;{\text{mm}}} \hfill \\ {Q^{\prime\prime}_{{\min }} ,} \hfill & {\sqrt {\left( {x - x_{0} } \right)^{2} + \left( {y - y_{0} } \right)^{2} } \ge {\text{1}}00\;{\text{mm}}} \hfill \\ \end{array} } \right.$$

where $Q^{\prime\prime}_{{\max }}$ and $Q^{\prime\prime}_{{\min }}$ are the maximum and minimum desired heat fluxes respectively, the parameters *x*_0_ and *y*_0_ are the coordinates of the center of the workpiece. When $\sqrt {\left( {x - x_{0} } \right)^{2} + \left( {y - y_{0} } \right)^{2} } {\text{ < 100 mm}}$, heat flux decreases from the center to the periphery. When $\sqrt {\left( {x - x_{0} } \right)^{2} + \left( {y - y_{0} } \right)^{2} } \ge {\text{100 mm}}$, heat flux maintains constant. $Q^{\prime\prime}_{{\max }}$ and $Q^{\prime\prime}_{{\min }}$ are set to be 67.0 kW/m^2^ and 1.2 kW/m^2^ respectively. The maximum gradient of the heat flux field is 1.1 MW/m^3^ which locates 100 mm from the center, and the desired power *P*_0_ equals 783 W.

**Figure 4:**
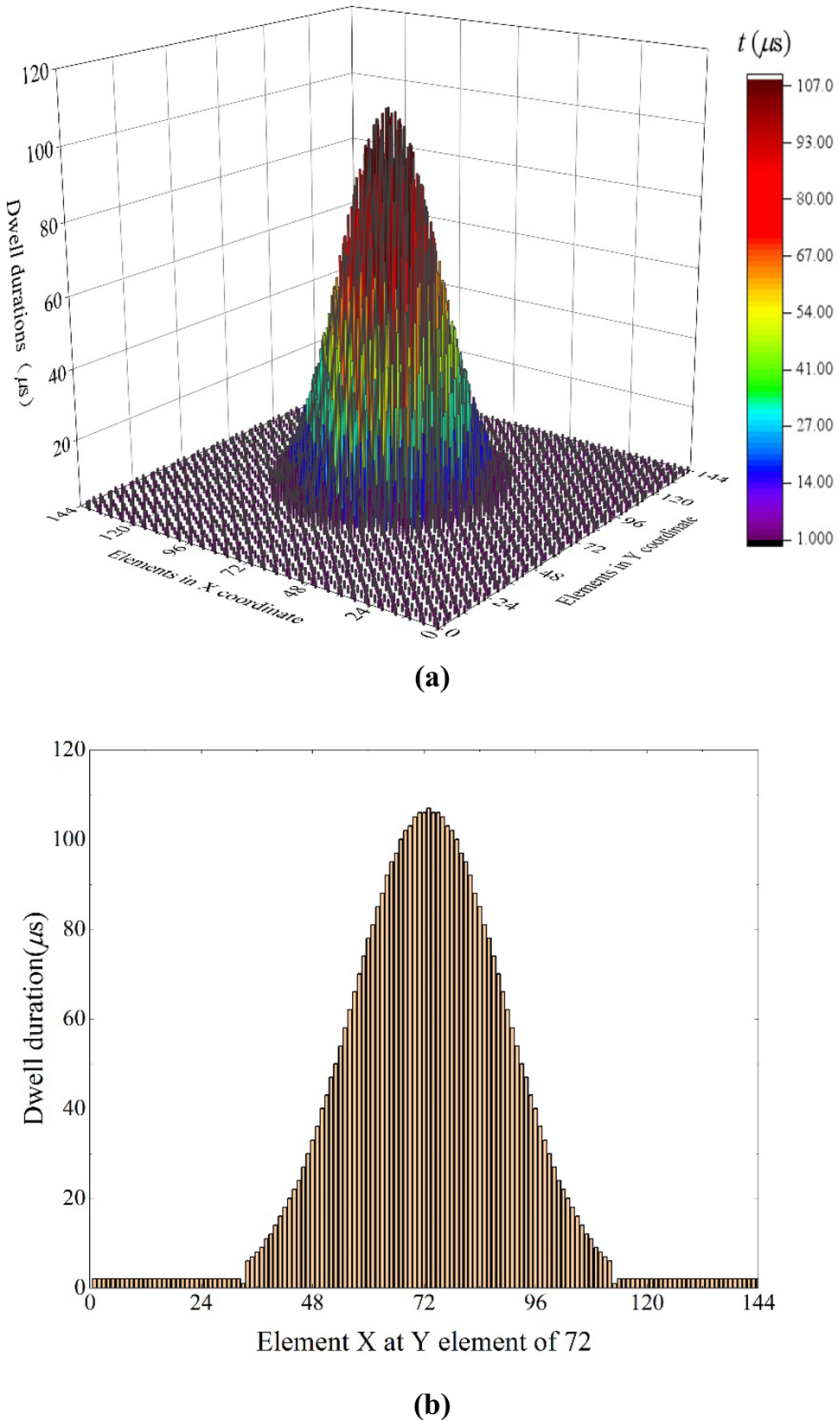
Dwell duration of the scanning strategy. (**a**) Results on the whole workpiece. (**b**) Results at Y element of 72.

The diameter of the beam spot is set as 5 mm during the experiment. By incorporating Eq. (9) into Eq. (4), the dwell duration can be obtained. The solution method of the equation set is to ensure that the maximum time-average scanned heat flux equals the maximum desired heat flux, and the accuracy is set to be lower than 5%. The results are shown in Fig. 4a,b. The dwell duration ranges from 1 to 107 μs, and the longest dwell duration locates at the center where the heat flux is the maximum. The shortest dwell duration locates 100 mm from the center, as there is a sharp heat flux decrease. The dwell duration of the regions over 100 mm away from the center is 2 µs. In the scanning process, to achieve the rapid scanning as short as 1 µs, current signal of high frequency is transferred to the Helmholtz deflection coils. Owing to the inductance of the Helmholtz coils, the shortest duration for the beam spot to jump between the elements is 1 µs.

**Figure 5:**
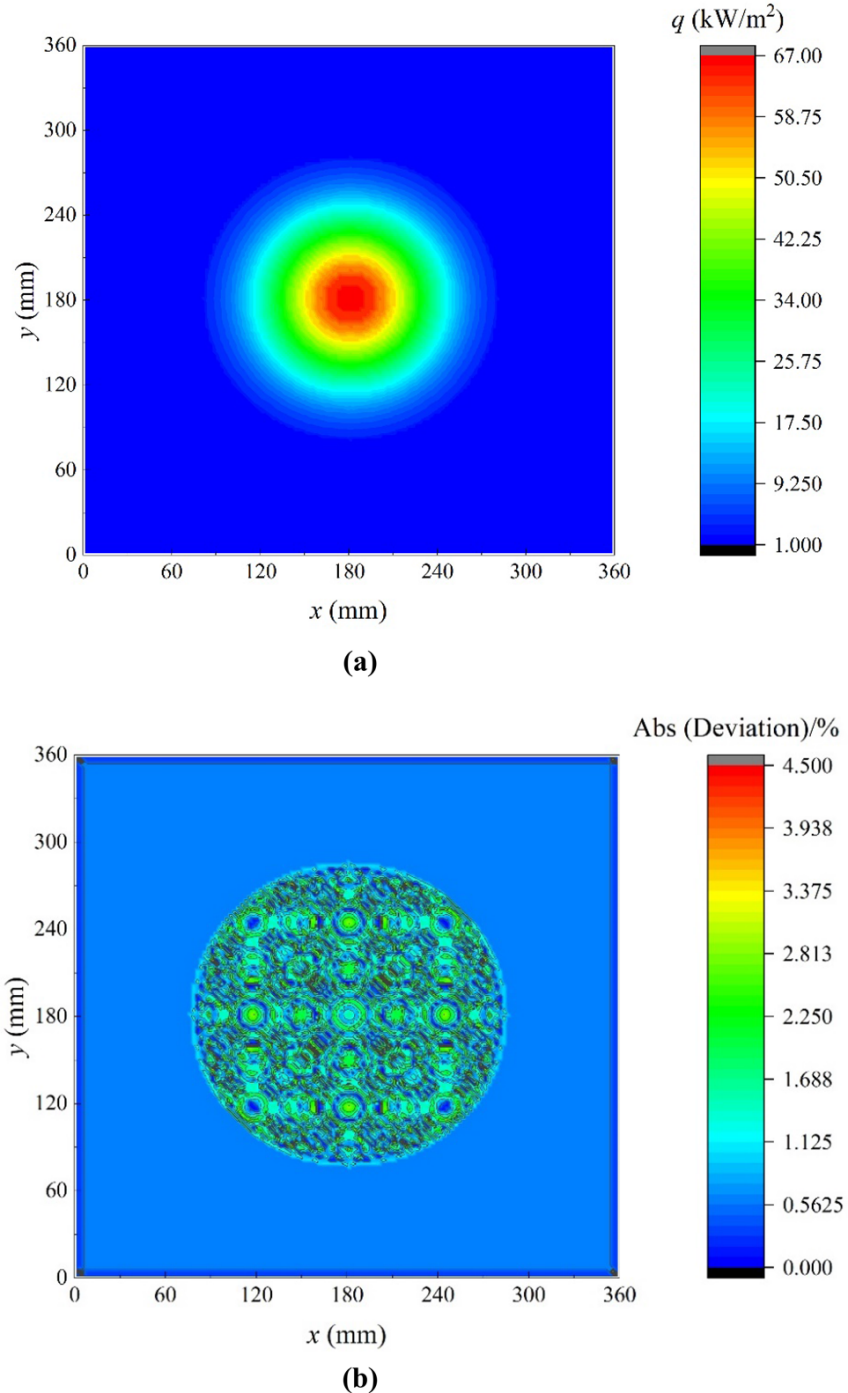
Heat flux distribution and relative deviation. (**a**) Time-average scanned heat flux. (**b**) Relative deviation of time-average scanned heat flux and desired heat flux.

According to the scanning strategy, the time-averaged scanned heat flux denoted as $\overline{{q^{\prime\prime}}} \left( {x,y} \right)$ was calculated as Eq. (2), and the results are shown in Fig. 5a. The time-averaged scanned heat flux is symmetric about the center and gradually decreases from the center to the periphery. The maximum time-average scanned heat flux is 67 kW/m^2^, and it equals to the maximum desired heat flux. The minimum time-average heat flux is 1.15 kW/m^2^, which is 4.2% smaller than the minimum desired heat flux. It can be seen that the distribution characteristics of the time-averaged scanned heat flux are consistent with those of the desired heat flux distribution. The relative deviation between the time-averaged scanned heat flux and the desired heat flux is shown in Fig. 5b. The maximum relative deviation is 4.5%, and the relative deviation in most areas is less than 3%. Therefore, the scanning strategy is theoretically verified.

### Verification of the variable-speed scanning method

“The scanning strategy” section illustrates the theoretical results of the method, in order to demonstrate it through experiments, the variable-speed scanning experiment was carried out by the electron beam thermal assessment equipment. The experiment included 3 stages with a total duration of 3000 s, and the duration, the beam current and the beam power of each stage are given in Table 3 while the acceleration voltage maintained 60 kV. Stage 1 was a preheating process lasted for 1250 s, and the corresponding current and power were 3.53 mA and 212 W respectively. The electron beam scanned at a constant speed. As the duration in a cycle is 0.2 s, the dwell duration is 10 μs in all the elements. Stage 2 and 3 were variable-speed scanning processes which lasted for 1010 s and 740 s respectively. The current in stage 2 was 5.90 mA, and the power was 0.45 *P*_0_ which equaled 354 W. The current in stage 3 was 11.40 mA, and the power was 0.87 *P*_0_ which equaled 684 W. The electron beam scanned at a variable speed, and the dwell duration is shown in Fig. 4. The diameter of the beam spot maintained 5 mm.

**Table 3:** The duration, beam power and beam power of the stages.

	Stage 1 (0–1250 s)	Stage 2 (1250–2260 s)	Stage 3 (2260–3000 s)
Current (mA)	3.53	5.90	11.40
Power(W)	212	354	684

The simulations of stage 2 and 3 are conducted through the heat transfer model. The powers are the same as that in the experiment, but the heat source is the corresponding time-average scanned heat flux. In stage 2, the heat source is described as 0.45 $\overline{{q^{\prime\prime}}} \left( {x,y} \right)$, the result of $\overline{{q^{\prime\prime}}} \left( {x,y} \right)$ is shown in Fig. 5a. In stage 3, the heat source is described as 0.87 $\overline{{q^{\prime\prime}}} \left( {x,y} \right)$.The simulation begins at 1250 s in stage 2, the initial temperature of the simulation is set as an average of T1–T3 at 1250 s.

**Figure 6:**
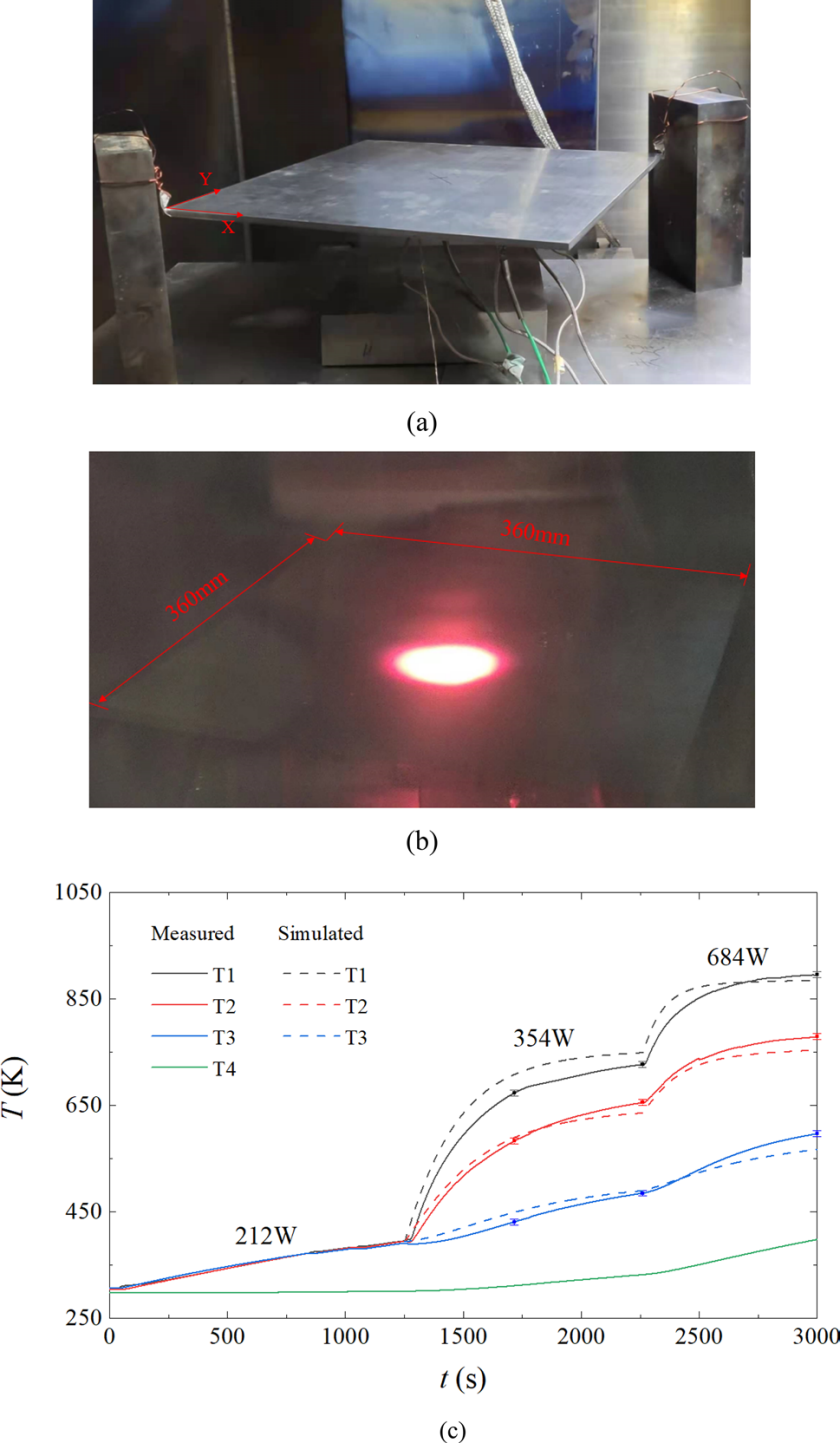
The photos for the experiments and the simulated and measured temperatures. (**a**) The workpiece hanging above the platform. (**b**)The experimental phenomenon of the non-uniform heating. (**c**) Simulated and measured temperatures of the TC4 workpiece.

The photos for the non-uniform heating experiment are shown in Fig. 6a,b. The workpiece hung above the platform which resulted in radiative boundary conditions for the workpiece, as Fig. 6a shows. The experimental phenomenon of a non-uniform heating is shown in Fig. 6b, and it was shot without illumination. The aim is to observe the central area of the workpiece which emitted bright red light owing to high temperature caused by long dwell duration of the electron beam. Figure 6c shows the measured and simulated temperature results of T1–T4 in the experiment. During the experiment, the temperature of T4 increases slowly and reaches 397 K in the end. It is used as *T*_plat_ in Eq. (8). The measured temperatures of T1–T3 were 303.7, 303.9 and 306.4 K respectively at the beginning of the experiment. In stage 1, the measured temperatures of T1–T3 increased at almost the same speed, and reach 390, 394 and 398 K individually in the end. In stage 2, the measured temperatures of T1 and T2 increases rapidly, while the temperature of T3 slightly increases, and there has been large temperature difference inside the TC4 workpiece. The simulated temperature of T1 are slightly higher than that the measured temperature of T1, and the measured temperatures and simulated temperatures fit well for T2 and T3. In stage 3, the temperatures of T1-T3 continue to increase, and the maximum temperature reaches 895 K. There are differences between the measured temperatures and the simulated temperatures, and the maximum difference is 30 K. There are some possible reasons, for example, the absorption efficiency of TC4 to the electron beam could change with temperature. In addition, it was observed that the beam spot deformed at the periphery of the workpiece due to defocusing, and the diameter of the beam spot changed in the deflection process. Overall, the experimental and simulated trends of temperature increment of points T1–T3 fit well, which verifies the method of variable-speed scanning of electron beam.

### Temperature fluctuation

“Verification of the variable-speed scanning method” section demonstrates the method, here, to investigate the instantaneous temperature increment and the amplitude of temperature fluctuation at various locations, a simulation of variable-speed scanning of the electron beam is conducted with the heat transfer model in “Experimental system” section. The heat source of the model in Eq. (6) is described by $q^{\prime\prime}_{{{\text{ij}}}} \left( {x_{{\text{j}}} ,y_{{\text{j}}} } \right)$. The electron beam scans according to the scanning strategy in “The scanning strategy” section. The calculation of the temperature fluctuation lasts for 1 s, i.e., 5 cycles.

**Figure 7:**
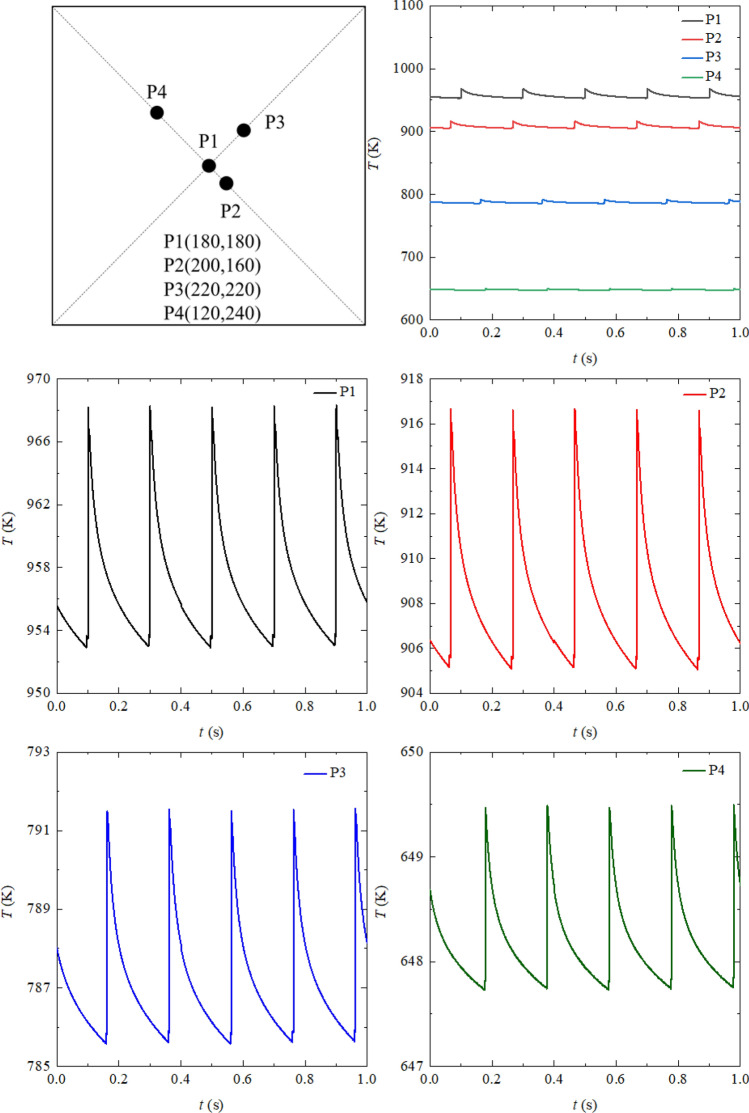
The simulated temperature fluctuation results of P1–P5 in 1 s.

The simulated temperature fluctuation results of points P1-P4 on the top surface are shown in Fig. 7. At the beginning of the calculation, the average temperatures of the points in a cycle have maintained constant, and the beam spot locates at the first element. The horizontal coordinates of the points are (180, 180), (200, 160), (220, 220) and (120, 240) respectively, and P1 is at the center of the top surface. The time-averaged scanned heat fluxes of the points are 67.0, 52.7, 25.6 and 7.7 kW/m^2^ respectively. The average temperatures of the points in a cycle range from 640 to 965 K, and they enlarge with the time-average scanned heat flux. The amplitude of temperature fluctuation is defined as

10 $$\Delta T = \left( {T_{{{\text{crest}}}} - T_{{{\text{trough}}}} } \right)/2$$

where *T*_crest_ and *T*_trough_ are the highest and the lowest temperature respectively in the scanning process. The instantaneous temperature increment is defined as 2 T$\Delta$. The amplitudes of the temperature fluctuation of the points are 7.2, 6.0, 3.1 and 1.0 K individually, and the maximum temperature increment is 14.4 K. The maximum amplitude of the temperature fluctuation is at P1, and the minimum amplitude of the temperature fluctuation appears at P4. It is attributed to that higher time-averaged scanned heat flux cause larger temperature fluctuation. Owing to different locations of the points, their temperatures reach the crest at different time, but the crests for all the points are at a constant interval of 0.2 s. It can be explained by the periodic scanning of the electron beam. The instantaneous temperature increment for all the points is lower than 15 K. Nevertheless, the thermal assessment temperature at the corresponding point over 950 K, hence it makes little thermal shock. The maximum amplitude of temperature fluctuation for all the points is merely 7.2 K, and it is not possible to cause thermal fatigue^31,32^.

Apart from the time-averaged scanned heat flux, to investigate the influence factors of temperature fluctuation and apply the thermal assessment method for more materials, a simulation of a 304 stainless steel workpiece heated by the scanning electron beam is conducted. The thermal model of the 304 stainless steel workpiece is constructed in the same way as that of the TC4 workpiece. The heat source of the model in Eq. (6) is described by $q^{\prime\prime}_{{{\text{ij}}}} \left( {x_{{\text{j}}} ,y_{{\text{j}}} } \right)$ either, but the absorption efficiency is assumed to be 0.82 according to the experiment^18^. The beam power, the diameter of the beam spot and the scanning strategy are the same as the TC4 workpiece discussed above. Density, thermal conductivity and specific heat of 304 stainless steel are given in Table 4^29^. The temperature fluctuation of points P1–P4 on the top surface of the 304 stainless steel workpiece are calculated. The results of the points in the 304 stainless steel are 4.0, 3.3, 1.7 and 0.6 K respectively. They are lower than that of the points in the TC4 workpiece with the same heat flux boundary, which can be attributed to the lower absorption efficiency and the larger volumetric heat capacity of 304 stainless steel. The thermal conductivity and the emissivity determine the average temperature but caused little effects to the temperature increment as the dissipated heat is negligible compared with the gained heat in the temperature increasing phase. Therefore, the amplitude of the temperature fluctuation increases with the time-averaged scanned heat flux and the absorption efficiency but decreases with the volumetric heat capacity.

**Table 4:** Thermal properties of 304 stainless steel.

T(K)	*ρ* (kg/m^3^)	*c*_p_ (J/kg K)	*k* (W/m K)	*ε*
298	8020	479	14.7	0.116
373	7982	500	15.8	0.126
473	7932	530	17.7	0.138
573	7882	540	18.8	0.150
673	7832	560	20.7	0.162
773	7782	570	21.4	0.174
873	7731	598	23.5	0.187
973	7681	600	24.6	0.200
1073	7631	620	25.8	0.211

**Figure 8:**
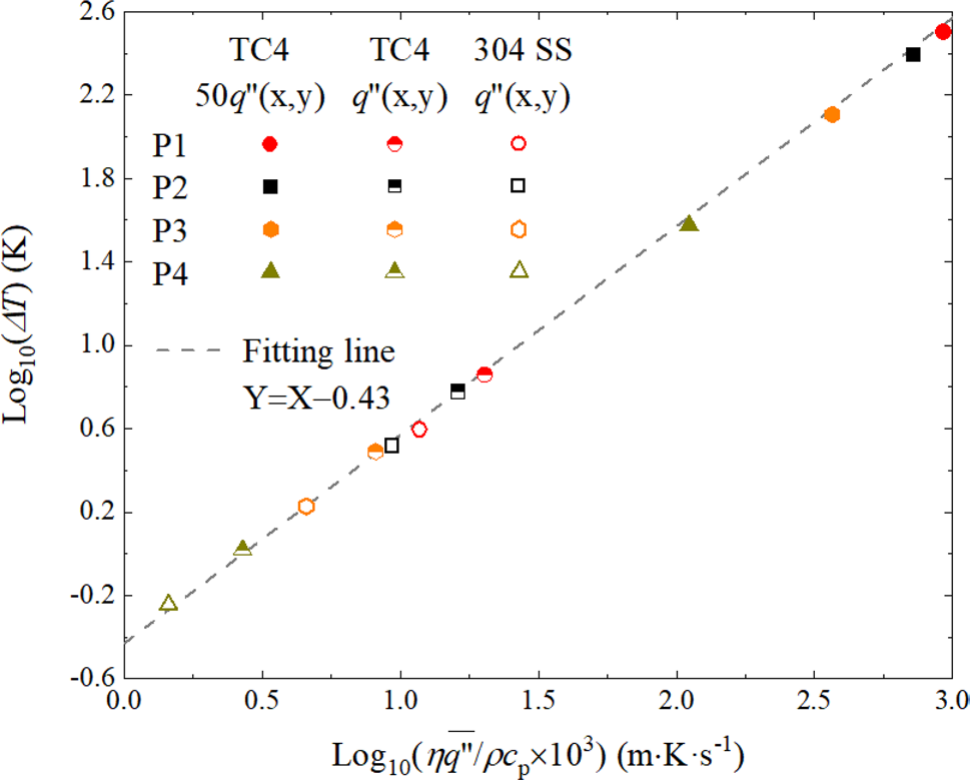
The relationship between the temperature fluctuation and $\overline{{q^{\prime\prime}}} /\rho c_{{\text{p}}}$ for the TC4 and the 304 stainless steel (304 SS) workpiece.

To investigate whether this novel method is applicable in thermal assessment of hypersonic aircrafts, a simulation of the TC4 workpiece with the heat source of $50q^{\prime\prime}_{{{\text{ij}}}} \left( {x_{{\text{j}}} ,y_{{\text{j}}} } \right)$ is conducted, and the corresponding maximum heat flux is 3.35 MW/m^2^, which is within the range of thermal assessment of high heat flux^5^. When the simulated temperatures exceed the melting point of TC4, the thermal properties are assumed to be equal to those at 1873 K in Table 1, and the emissivity is assumed to be equal to those at 1183 K in Table 2. The temperature fluctuation of points P1–P4 on the top surface of the TC4 workpiece are calculated, and the results are 328.1, 247.2, 127.7 and 37.6 K respectively. The relationship between the amplitude of the temperature fluctuation and $\eta \overline{{q^{\prime\prime}}} /\rho c_{{\text{p}}}$ for the workpieces are shown in Fig. 8. The simulated results are processed in a logarithmic form, and it is found that $\log _{{10}} \left( {\Delta T} \right)$ and $\log _{{10}} \left( {\eta \overline{{q^{\prime\prime}}} /\rho c_{{\text{p}}} \times 10^{3} } \right)$ can be fitted to the line of $Y = X - 0.43$, which means $\Delta T$ is nearly proportional to $\eta \overline{{q^{\prime\prime}}} /\rho c_{{\text{p}}}$ for the points in both the 304 stainless steel and TC4 workpiece. Therefore, when the temperature fluctuation distribution at one point is obtained, the amplitude of temperature fluctuation at various locations can be predicted for different materials at different heat flux conditions below 3.35 MW/m^2^. In terms of the large amplitude of temperature fluctuation with high heat flux, the means to reduce it include increasing the scanning frequency and decrease the number of the elements.

## Conclusions

A novel method to apply a variable-speed scanning electron beam to realize non-uniform precise heat flux boundary conditions is proposed. The maximum scanning frequency of the Helmholtz deflecting coils determines the scanning range and the scanning cycle. Based on the conservation of energy, the scanning method is constructed. The method is verified when applied in a heat flux field of large gradient and the solution deviation is less than 5%. The corresponding scanning strategy can be provided. The feasibility of the method is verified by the experiment. The instantaneous temperature increment should be considered to avoid serious thermal shock and thermal fatigue. The amplitude of temperature fluctuation is proportional to $\eta \overline{{q^{\prime\prime}}} /\rho c_{{\text{p}}}$ below the heat flux of 3.35 MW/m^2^, thus the applicability of the thermal assessment method for other materials can be predicted before testing.

## Data Availability

The data that support the experimental results are available from the corresponding author upon the reasonable request.
